# Statins for atherosclerotic cardiovascular disease prevention in people living with HIV in Thailand: a cost‐effectiveness analysis

**DOI:** 10.1002/jia2.25494

**Published:** 2020-06-19

**Authors:** David C Boettiger, Anthony T Newall, Pairoj Chattranukulchai, Romanee Chaiwarith, Suwimon Khusuwan, Anchalee Avihingsanon, Andrew Phillips, Eran Bendavid, Matthew G Law, James G Kahn, Jeremy Ross, Sergio Bautista‐Arredondo, Sasisopin Kiertiburanakul

**Affiliations:** ^1^ Kirby Institute UNSW Sydney Sydney NSW Australia; ^2^ Institute for Health Policy Studies University of California San Francisco CA USA; ^3^ The School of Public Health and Community Medicine UNSW Sydney Sydney NSW Australia; ^4^ Cardiac Center Chulalongkorn University Chulalongkorn Memorial Hospital King Bangkok Thailand; ^5^ Research Institute for Health Sciences Chiang Mai University Chiang Mai Thailand; ^6^ Chiangrai Prachanukroh Hospital Chiang Rai Thailand; ^7^ The Thai Red Cross AIDS Research Centre and Faculty of Medicine Chulalongkorn University Bangkok Thailand; ^8^ Institute for Global Health University College London United Kingdom; ^9^ Center for Health Policy and the Center for Primary Care and Outcomes Research Stanford University Stanford CA USA; ^10^ TREAT Asia/amfAR–Foundation for AIDS Research Bangkok Thailand; ^11^ Center for Health Systems Research National Institute of Public Health Cuernavaca Mexico; ^12^ Faculty of Medicine Ramathibodi Hospital Mahidol University Bangkok Thailand

**Keywords:** HIV, cardiovascular disease, statin, cost‐effectiveness, Thailand, antiretroviral therapy

## Abstract

**Introduction:**

People living with HIV (PLHIV) have an elevated risk of atherosclerotic cardiovascular disease (CVD) compared to their HIV‐negative peers. Expanding statin use may help alleviate this burden. However, the choice of statin in the context of antiretroviral therapy is challenging. Pravastatin and pitavastatin improve cholesterol levels in PLHIV without interacting substantially with antiretroviral therapy. They are also more expensive than most statins. We evaluated the cost‐effectiveness of pravastatin and pitavastatin for the primary prevention of CVD among PLHIV in Thailand who are not currently using lipid‐lowering therapy.

**Methods:**

We developed a discrete‐state microsimulation model that randomly selected (with replacement) individuals from the TREAT Asia HIV Observational Database cohort who were aged 40 to 75 years, receiving antiretroviral therapy in Thailand, and not using lipid‐lowering therapy. The model simulated each individual’s probability of experiencing CVD. We evaluated: (1) treating no one with statins; (2) treating everyone with pravastatin 20mg/day (drug cost 7568 Thai Baht ($US243)/year) and (3) treating everyone with pitavastatin 2 mg/day (drug cost 8182 Baht ($US263)/year). Direct medical costs and quality‐adjusted life‐years (QALYs) were assigned in annual cycles over a 20‐year time horizon and discounted at 3% per year. We assumed the Thai healthcare sector perspective.

**Results:**

Pravastatin was estimated to be less effective and less cost‐effective than pitavastatin and was therefore dominated (extended) by pitavastatin. Patients receiving pitavastatin accumulated 0.042 additional QALYs compared with those not using a statin, at an extra cost of 96,442 Baht ($US3095), giving an incremental cost‐effectiveness ratio of 2,300,000 Baht ($US73,812)/QALY gained. These findings were sensitive to statin costs and statin efficacy, pill burden, and targeting of PLHIV based on CVD risk. At a willingness‐to‐pay threshold of 160,000 Baht ($US5135)/QALY gained, we estimated that pravastatin would become cost‐effective at an annual cost of 415 Baht ($US13.30)/year and pitavastatin would become cost‐effective at an annual cost of 600 Baht ($US19.30)/year.

**Conclusions:**

Neither pravastatin nor pitavastatin were projected to be cost‐effective for the primary prevention of CVD among PLHIV in Thailand who are not currently using lipid‐lowering therapy. We do not recommend expanding current use of these drugs among PLHIV in Thailand without substantial price reduction.

## 1 Introduction

People living with HIV (PLHIV) have an elevated risk of atherosclerotic cardiovascular disease (CVD) compared to their HIV‐negative peers [1]. This is only partially explained by the high prevalence of cardiovascular risk factors among PLHIV. In a landmark study of 82,459 US veterans, those who were HIV‐positive had a 48% increased risk of incident myocardial infarction (MI) compared with HIV‐negative participants, even after adjusting for well‐known risk factors, comorbidities and substance use [2]. Similar studies have also found a small, but significant increase in ischaemic stroke incidence associated with HIV infection [3, 4, 5].

The increased CVD risk associated with HIV may be mediated by the virus itself, past or present immunodeficiency, adverse effects of antiretroviral therapy (ART), deficiencies in cardiovascular care in PLHIV, or a combination of these factors [6]. As ART is often initiated at an advanced stage of HIV in low‐ and middle‐income countries [7] and untreated HIV is associated with an increased risk of CVD [8], it is possible that the risk is further exacerbated in settings where resources are limited [9, 10].

Statins reduce atherosclerotic CVD risk primarily by lowering LDL cholesterol levels [11]. They also have anti‐inflammatory properties that may enhance their effectiveness in PLHIV [12]. In a recent survey of HIV clinics in low‐ and middle‐income Asian countries (including Thailand), 94% reported that patients could access statins through the clinic or the same facility as the clinic [13]. Current Thai guidelines recommend lipid‐lowering therapy be initiated among PLHIV with a 10‐year risk of CVD greater than 10%, consistent with general population guidelines [14]. However, studies among the general population in Thailand and other middle‐income countries suggest statin use could be cost‐effective for those at lower risk of CVD [15, 16, 17]. It is uncertain whether this is likely to extend to PLHIV, despite the elevated risk of CVD associated with HIV infection, as statin use in the context of ART is complicated by the risk of drug interactions leading to intolerance or reduced efficacy. With concomitant protease inhibitor use, simvastatin and lovastatin are contraindicated, whereas atorvastatin and rosuvastatin require modified dosing [18]. With concomitant efavirenz or etravirine use, statins may require dose modification [18].

Pravastatin and pitavastatin are preferred agents among PLHIV because they improve cholesterol levels and reduce immune activation without interacting substantially with ART [19, 20, 21, 22]. Although generic pravastatin formulations are available, they tend to be more expensive than other generic statin formulations. Pitavastatin is a newer statin and in many settings is more expensive than pravastatin, however, current evidence suggests it produces greater improvements in cholesterol levels than pravastatin among PLHIV [19].

Given these trade‐offs, we evaluated the cost‐effectiveness of pravastatin and pitavastatin for the primary prevention of CVD among PLHIV in Thailand who are not currently using lipid‐lowering therapy. We believe this is the first study to assess the cost‐effectiveness of expanded statin use among PLHIV in Thailand.

## 2 Methods

### 2.1 Study population

We used individual patient data from all Thai sites contributing to the TREAT Asia HIV Observational Database (TAHOD), the updated Data collection on Adverse Effects of Anti‐HIV Drugs (D:A:D) CVD risk equation, and published literature to estimate medical costs and quality‐adjusted life‐years (QALYs) among adult PLHIV in Thailand. TAHOD is an ongoing collaboration of 21 HIV clinics in the Asia‐Pacific region that is part of the International epidemiology Databases to Evaluate AIDS Asia‐Pacific [23]. Participating clinics follow local guidelines and regulations regarding patient consent and ethics review. Our study population included patients enrolled in TAHOD at one of the four Thai sites involved (Ramathibodi Hospital, Bangkok; HIV‐NAT Research Collaboration/Thai Red Cross AIDS Research Centre, Bangkok; Research Institute for Health Sciences, Chiang Mai; and Chiangrai Prachanukroh Hospital, Chiang Rai) who had documentation of at least one clinic visit on or after 1 January 2013 and who, at their last documented clinic visit, were aged 40 to 75 years, had no history of CVD, were not using lipid‐lowering therapy, had been using ART for at least six months, and had a CD4 cell count> 100 cells/mm^3^. Stable ART was included as a selection criterion as this should be prioritized by PLHIV over CVD risk management. Table 1 further characterizes the 917 PLHIV included in our study population.

**Table 1:** Study population characteristics at beginning of simulation

Characteristic	N = 917	
Sex	Male	442 (48.2)
Age, years	Median (IQR)	48.6 (44.6, 54.6)
Mode of HIV exposure	Heterosexual	840 (91.6)
	Homosexual	53 (5.8)
	Intravenous drug use	13 (1.4)
	Other	11 (1.2)
Hepatitis C antibody status	Positive	55 (6.0)
Hepatitis B surface antigen status	Positive	90 (9.8)
Family history of CVD	Yes	84 (9.2)
Diabetic	Yes	60 (6.5)
Current smoker	Yes	133 (14.5)
Ever smoked	Yes	327 (35.7)
Systolic blood pressure, mmHg	Median (IQR)	124 (115, 135)
Using antihypertensive medication	Yes	105 (11.5)
Total cholesterol, mmol/L	Median (IQR)	5.0 (4.3, 5.6)
HDL cholesterol, mmol/L	Median (IQR)	1.3 (1.1, 1.6)
LDL cholesterol, mmol/L	Median (IQR)	3.3 (2.7, 3.9)
CD4 cell count, cells/mm^3^	Median (IQR)	555 (419, 712)
D:A:D risk score, 5‐year risk of CVD	≤1%	227 (24.8)
	>1% to 5%	556 (60.6)
	>5%	134 (14.6)

All values are n (%N) unless otherwise specified. CVD, cardiovascular disease; IQR, interquartile range; D:A:D, data collection on Adverse Effects of Anti‐HIV Drugs study.

### 2.2 Model structure

We developed a discrete‐state microsimulation model that randomly selected (with replacement) 10,000 patients from our study population and simulated their experience over time. The model assumed the Thai healthcare sector perspective and applied a 20‐year time horizon. Patients started in the healthy state and were at risk of coronary intervention without an MI, MI, ischaemic stroke, haemorrhagic stroke, cardiovascular death or non‐CVD death. Coronary interventions included coronary artery bypass graft (CABG) and percutaneous coronary intervention (PCI). Further detail is provided in the Supplementary Material, including Figure S1 which presents a schematic of the core model structure. Key model parameters are detailed in Table 2.

**Table 2:** Key model parameters

Parameter	Base case (range for sensitivity)	Source
Probabilities		
CVD risk factors		
Probability of CVD event (D:A:D equation)	Varies by individual^a^	[24]
Annual probability of developing diabetes	Varies by age and sex^b^	[25]
Annual probability of smoking cessation^c^	Varies by age^b^	[26]
Increase in systolic blood pressure per year of age	Varies by age and sex^b^	[27]
Myocardial Infarction		
Probability of CVD event being fatal/non‐fatal MI	0.488 (0.450 to 0.520)	[24]
Probability of CABG after MI^c^	0.031 (0.024 to 0.039)	[28]
Probability of PCI after MI^c^	0.288 (0.268 to 0.308)	[28]
Probability of MI being fatal^c^	0.177 (0.161 to 0.195)	[28]
Stroke		
Probability of CVD event being fatal/non‐fatal stroke	0.292 (0.250 to 0.320)	[24]
Probability of stroke being ischemic^c^	0.693 (0.690 to 0.700)	[29]
Probability of ischemic stroke being fatal^c^	0.284 (0.236 to 0.335)	[30]
Probability of hemorrhagic stroke being fatal^c^	0.484 (0.358 to 0.613)	[30]
CVD intervention (without prior MI/stroke)		
Probability of CVD event being an intervention	0.163 (0.140 to 0.190)	[24]
Probability of intervention being CABG^c^	0.241 (0.185 to 0.303)	[28]
Probability of intervention being PCI^c^	0.759 (0.697 to 0.815)	[28]
Probability of MI after CABG^c^	0.100 (0.050 to 0.300)	[31]
Probability of MI after PCI^c^	0.041 (0.036 to 0.048)	[32]
Death		
Probability of CVD event being other CVD death	0.044 (0.030 to 0.060)	[24]
Hazard of other CVD death for past MI/stroke vs no past MI/stroke	2.000 (1.000 to 3.000)	Assumption
Probability of non‐CVD death	Varies by age, sex and CD4^d^	[33]
Recurrent events		
Probability of recurrent MI^c^	Varies by age, sex and time since last MI^d^	[34, 35]
Probability of CABG after recurrent MI^c^	0.031 (0.024 to 0.039)	[28]
Probability of PCI after recurrent MI^c^	0.288 (0.268 to 0.308)	[28]
Probability that recurrent MI is fatal^c^	0.217 (0.109 to 0.364)	[36]
Probability of recurrent ischemic stroke^c^	Varies by time since last stroke^d^	[30, 37, 38, 39]
Probability that recurrent ischemic stroke is fatal^c^	0.270 (0.140 to 0.420)	[37]
Probability of recurrent hemorrhagic stroke in first year after initial^c^	0.057 (0.015 to 0.409)	[37]
Probability of recurrent hemorrhagic stroke in subsequent years^c^	Varies by individual^e^	[37]
Probability that recurrent hemorrhagic stroke is fatal^c^	0.430 (0.070 to 0.930)	[37]
Probability of ischemic stroke after MI^c^	Varies by gender and time since MI^d^	[37, 38, 39, 40]
Probability that ischemic stroke after MI is fatal^c^	0.270 (0.140 to 0.420)^f^	Assumption
Probability of MI after stroke^c^	Varies by age, gender and time since stroke^d^	[35, 38, 41]
Probability that MI after stroke is fatal^c^	0.217 (0.109 to 0.364)^g^	Assumption
Probability of hemorrhagic stroke after MI	Varies by individual^e^	Assumption
Probability of hemorrhagic stroke after ischemic stroke	Varies by individual^e^	Assumption
Probability of hemorrhagic stroke after MI or ischemic stroke being fatal^c^	0.484 (0.358 to 0.613)^h^	Assumption
Efficacy and safety of pravastatin and pitavastatin		
Reduction in total cholesterol associated with pravastatin 20 mg, %	13.7 (2.2 to 25.2)	[19]
Reduction in total cholesterol associated with pitavastatin 2 mg, %	19.1 (6.9 to 31.3)	[19]
Increase in HDL cholesterol associated with pravastatin 20 mg, %	7.2 (0.0 to 22.6)	[19]
Increase in HDL cholesterol associated with pitavastatin 2 mg, %	8.9 (0.0 to 26.4)	[19]
Additional reduction in CVD risk associated with statin use (i.e. due to factors other than lipid change), %	0.0 (0.0 to 30.0)	Assumption
Hazard ratio of hemorrhagic stroke for statin use vs no statin^c^	1.0001 (1.0000 to 1.0002)	[11]
Costs, 2018 Thai Baht		
HIV management	59,856 (29,929 to 89,784)	[42, 43]
Non‐fatal MI medical management^c^	35,441 (17,721 to 53,162)	[44]
PCI^c^	215,765 (107,882 to 323,647)	[44]
CABG^c^	316,475 (158,238 to 474,714)	[44]
Non‐fatal MI management – first year post‐MI^c^	62,245 (34,974 to 143,252)	[45]
Non‐fatal MI management – after first year post‐MI^c^	17,780 (8890 to 26,670)	[45]
Fatal MI^c^	221,915 (81,878 to 356,072)	[45]
Non‐fatal ischemic stroke hospitalization^c^	26,668 (23,497 to 29,820)	[17, 46]
Non‐fatal ischemic stroke management – first year post‐stroke^c^	42,435 (39,284 to 45,587)	[17, 46]
Non‐fatal ischemic stroke management – after first year post‐stroke^c^	10,932 (8746 to 13,119)	[17]
Fatal ischemic stroke^c^	54,671 (43,737 to 65,606)	[17]
Non‐fatal hemorrhagic stroke hospitalization^c^	26,668 (23,497 to 29,820)^i^	Assumption
Non‐fatal hemorrhagic stroke management – first year post‐stroke^c^	42,435 (39,284 to 45,587)^i^	Assumption
Non‐fatal hemorrhagic stroke management – after first year post‐stroke^c^	10,932 (8746 to 13,119)^i^	Assumption
Fatal hemorrhagic stroke^c^	54,671 (43,737 to 65,606)^i^	Assumption
Other cardiovascular death^c^	221,915 (81,878 to 356,072)^j^	Assumption
Statin‐associated diabetes, average cost/individual taking statin/year^c^	2.30 (1.70 to 3.70)	[47, 48]
Statin‐associated myopathy, average cost/individual taking statin/year^c^	0.05 (0.02 to 0.08)	[49, 50]
Pravastatin 20 mg, 12‐month supply	7568 (3784 to 11,352)	[51]
Pitavastatin 2 mg, 12‐month supply	8182 (4091 to 12,273)	[51]
Blood lipid test	660 (495 to 825)	[15]
Utilities		
Weights		
No history of CVD	1.0000	Assumption
History of MI^c^	0.9510 (0.9280 to 0.9690)	[52]
History of ischemic stroke^c^	0.6840 (0.5630 to 0.7940)	[52]
History of hemorrhagic stroke^c^	0.6840 (0.5630 to 0.7940)	[52]
History of MI and ischemic stroke^c^	0.6505 (0.5225 to 0.7694)	[52]
History of MI and hemorrhagic stroke^c^	0.6505 (0.5225 to 0.7694)	[52]
Quality‐of‐life decrements		
PCI^c^	0.0061 (0.0040 to 0.0087)	[52]
CABG^c^	0.0128 (0.0084 to 0.0184)	[52]
Acute MI^c^	0.0076 (0.0051 to 0.0106)	[52]
Acute ischemic stroke^c^	0.0242 (0.0158 to 0.0335)	[52]
Acute hemorrhagic stroke^c^	0.0242 (0.0158 to 0.0335)	[52]
Diabetes, average toll/individual taking statin/year^c^	0.00005 (0.00003 to 0.00007)	[47, 52]
Myopathy, average toll/individual taking statin/year^c^	0.0000010 (0.0000007 to 0.0000012)	[49, 52]
Daily statin administration/pill burden^c^	0.00000 (0.00000 to 0.00384)	[53]
Discounting and time horizon		
Annual discount rate (applied to costs and benefits)	0.03 (0.00 to 0.05)	[54]
Time horizon, years	20 (10 to 30)	[54]

a D:A:D equation uses age, sex, diabetes status, family history of CVD, current and past smoking status, total cholesterol, HDL cholesterol, systolic blood pressure, and CD4 cell count to calculate CVD risk

b see Tables S1, S2, S3

c based on general population or high‐income setting

d see Figures S2, S3, S4, S5, S6

e same as probability of incident hemorrhagesame as probability of incident hemorrhagic stroke calculated with D:A:D equation.ic stroke calculated with D:A:D equation;

f same as probability of recurrent ischemic stroke being fatal

g same as probability of incident hemorrhagic stroke calculated with D:A:D equation

h same as probability of incident hemorrhagic stroke being fatal

i as for ischemic stroke hospitalization/management

j as for fatal MI. CVD, cardiovascular disease; CABG, coronary artery bypass graft; PCI, percutaneous coronary intervention; MI, myocardial infarction.

At the start of each annual cycle, the model estimated an individual’s probability of transitioning from the healthy state to one of the CVD states based on their D:A:D CVD risk score [24, 55]. The D:A:D equation is the only well‐validated HIV‐specific CVD risk equation and is recommended for PLHIV by the American Heart Association [56]. It has been shown to produce similar estimates to the Ramathibodi‐Electricity Generating Authority of Thailand (Rama‐EGAT) CVD risk equation [57] among PLHIV in Thailand [58]. We used the reduced D:A:D CVD risk equation (which is based on patient age, sex, diabetes status, family history of CVD, current smoking status, past smoking status, total cholesterol, HDL cholesterol, systolic blood pressure and CD4 count) rather than the full equation (which also includes ART) because the reduced model is recommended for patients exposed to ART for more than five years [24]. Individual CVD risk scores were calculated using patient data, adding one year of age for each cycle, assuming age‐ and sex‐specific changes in systolic blood pressure [27] and rates of diabetes [25], and age‐specific rates of smoking cessation [26]. All other variables used to calculate CVD risk were kept constant over time.

As the D:A:D risk score calculates five‐year probability of CVD, we converted scores to rates, divided them by five and converted to one‐year probabilities. Since the risk score defines CVD as a composite of coronary intervention, MI, stroke (ischaemic or haemorrhagic) or other cardiovascular death, we apportioned the calculated risk into individual event types based on the proportions reported in Friis‐Moller *et al*. [24] Each individuals risk of non‐CVD death was estimated by subtracting their calculated risk of CVD death from their age, sex and CD4 count specific risk of all‐cause mortality [33]. Recurrent event probabilities (for example, the probability of a second MI or the probability of an MI after a prior ischaemic stroke) were mainly based on published estimates for the general population in high‐income countries due to a lack of HIV‐specific data or data from low‐ and middle‐income countries; we did not use the D:A:D CVD risk score or a HIV‐specific hazard ratio as current evidence suggests that risk factors for primary CVD differ substantially from those of recurrent CVD [34, 59, 60, 61]. Individuals accumulated costs and benefits up until their death or the time horizon, whichever came first.

### 2.3 Treatment strategies

We evaluated three treatment strategies in our base‐case analysis: (1) treating none of the study population with a statin; (2) treating the entire study population with pravastatin 20 mg/day and (3) treating the entire study population with pitavastatin 2 mg/day. We assumed patients using pravastatin and pitavastatin would exhibit sufficient adherence to achieve the same improvements in total cholesterol and HDL cholesterol observed in a recent clinical trial among PLHIV [19]. This trial, which primarily recruited participants of non‐Asian ethnicity, used doses of 40 mg/day for pravastatin and 4 mg/day for pitavastatin. However, we assumed lower doses would achieve similar efficacy in Asian patients, as has been shown for other statins [62]. Total and HDL cholesterol improvements were used to quantify efficacy, despite statins primarily reducing CVD risk via lowering LDL cholesterol, because these are the cholesterol variables included in the D:A:D CVD risk equation. In our base‐case analysis, we assumed pravastatin would reduce total cholesterol by 13.7% and increase HDL cholesterol by 7.2%, whereas pitavastatin would reduce total cholesterol by 19.1% and increase HDL cholesterol by 8.9% [19]. We assumed statin therapy only reduced CVD risk by improving cholesterol levels. However, in sensitivity analyses, we assumed additional CVD preventative efficacy to account for the possibility that the anti‐inflammatory properties of statins may provide additional benefit among PLHIV [12].

We did not assume statins prevent any non‐CVD outcomes as the current literature on this topic is inconclusive [63]. Since statins have been associated with an increased risk of haemorrhagic stroke [11], diabetes [47] and myopathy [49], we assumed hazard ratios and costs for these adverse events consistent with literature from the general population (see Table 2). Current evidence suggests there is little difference between statin types in terms of their adverse event profile [64].

### 2.4 Cost and quality‐of‐life estimates

Health‐related costs and quality‐of‐life (health state utility) adjustments were assigned to each clinical event and health state in annual cycles. We included all direct medical costs regardless of who paid for them. Cost estimates obtained from earlier years were inflated to 2018 Thai Baht equivalents using the World Bank Gross Domestic Product deflator [65] The cost of HIV management was based on estimates by Over *et al*. [42] and rates of second‐line ART use from TAHOD [43]. Drug costs across HIV clinics in Thailand are variable, however, we are not aware of any formal analysis. To best account for this uncertainty, we have used the 2018 unit prices published by Thailand’s National Drug System Development Committee [51] and varied these estimates widely in sensitivity analyses. Other costs were based on published estimates for the general population (see Table 2). Most quality‐of‐life adjustments were based on data from the 2017 Global Burden of Disease study [52]. Since patients using ART are already required to take at least one daily pill, we assumed that remembering to take a daily statin and the inconvenience of doing so (pill burden) was not associated with a quality‐of‐life decrement. Earlier studies among the general population have similarly assumed regular statin use is not associated with a pill burden [53]. Future costs and benefits were discounted at 3% per year [54].

### 2.5 Outcomes

The primary outcome was the incremental cost‐effectiveness ratio (ICER; defined as the cost per QALY gained). The threshold for an intervention being deemed cost‐effective (willingness‐to‐pay threshold) was defined as an ICER below 160,000 Baht ($US5315), as recommended by the Health Intervention and Technology Assessment Program, Ministry of Public Health, Thailand [66]. Our secondary outcomes included incremental QALYs gained, incremental costs incurred, incremental life‐years gained and the incremental cost per life‐year gained.

### 2.6 Sensitivity analyses

We used sensitivity analyses to evaluate the robustness of our results to uncertainty in key input parameters. In deterministic sensitivity analyses we varied one or two input parameters at a time while holding others constant at their base‐case estimates. In probabilistic sensitivity analyses we varied multiple input parameters across prespecified distributions over 500 iterations. Beta distributions were used for utilities and event probabilities, and log‐normal distributions were used for hazard ratios, safety and efficacy measures, and costs.

### 2.7 Scenario analyses

In addition to our sensitivity analyses, we investigated the following scenarios to explore different methodological choices:

1. Restricting the intervention to PLHIV at> 1% risk of CVD in the next five years (as defined by the D:A:D equation). In this scenario and in scenario 2 described below, we assumed an annual cost of 660 Baht ($US21.20)/person for blood lipid testing while an individual’s CVD risk score remained below the threshold for starting a statin [15]. We also performed analyses without this assumption to account for the availability of CVD risk equations that do not use blood lipid test results [57].
2. Restricting the intervention to PLHIV at> 5% risk of CVD in the next five years (as defined by the D:A:D equation).
3. Using the Rama‐EGAT equation to calculate MI and ischaemic stroke risk in place of the D:A:D equation. The Rama‐EGAT equation was developed using data from a study of 3499 HIV‐negative Thais [67] and has been validated in the general Thai population [68]. It calculates CVD risk based on age, sex, diabetes status, current smoking status, total cholesterol, HDL cholesterol and systolic blood pressure.[57] Although CVD risk equations based on the general population often underestimate CVD risk in PLHIV [69, 70], the Rama‐EGAT and D:A:D equations have been shown to produce similar estimates of CVD risk in PLHIV in Thailand [58].

### 2.8 Software

Data management and statistical analysis was conducted using SAS 9.4 (SAS Institute Inc, Cary, NC, USA). Modelling was performed in TreeAge Pro 2019 Version R1.0 (TreeAge Software, Williamstown, MA, USA).

## 3 Results

### 3.1 Base‐case analysis

Modelled incidence rates for MI, ischaemic stroke and fatal CVD among the no statin group were 4.7, 2.2 and 2.5 per 1000 person‐years respectively. These figures are consistent with observed rates of CVD reported for similarly aged participants in TAHOD [71]. The all‐cause mortality rate in the no statin group was 33.9 per 1000 person‐years and, over the next 20 years, patients were projected to accumulate a discounted average of 12.211 QALYs, 12.266 life‐years and 755,076 Baht ($US24,232) in direct medical costs (Table 3).

**Table 3:** Incremental cost‐effectiveness of pravastatin and pitavastatin for primary prevention of CVD among PLHIV over 20‐year time horizon

Intervention	Total cost, Baht	Statin cost, Baht	Lipid testing cost, Baht	MI^a^	Ischaemic stroke^a^	Fatal CVD^a^	All‐cause mortality^a^	Life‐years	QALYs	Incremental cost, Baht	Incremental life‐years gained	Incremental QALYs gained	Baht/life‐year gained^b^	ICER, Baht/QALY gained^b^
Base‐case														
No statin	755,076	0	0	4.7	2.2	2.5	33.9	12.266	12.211	–	–	–	–	–
Pravastatin 20 mg	844,370	90,517	0	3.9	1.9	2.2	33.6	12.290	12.241	Dominated (extended)				
Pitavastatin 2 mg	851,518	98,099	0	3.7	1.7	2.1	33.5	12.300	12.253	96,442	0.034	0.042	2,862,000	2,300,000
Scenario 1) Treat those with> 1% risk of CVD in the next five years														
No statin	755,076	0	0	4.7	2.2	2.5	33.9	12.266	12.211	–	–	–	–	–
Pravastatin 20 mg	837,599	83,237	1125	3.9	1.9	2.2	33.6	12.288	12.239	Dominated (extended)				
Pitavastatin 2 mg	844,207	90,210	1125	3.7	1.8	2.1	33.5	12.297	12.251	89,131	0.031	0.039	2,845,000	2,270,000
Scenario 2) Treat those with> 5% risk of CVD in the next five years														
No statin	755,076	0	0	4.7	2.2	2.5	33.9	12.266	12.211	–	–	–	–	–
Pravastatin 20 mg	778,141	22,540	6004	4.2	2.1	2.3	33.7	12.286	12.234	Dominated (extended)				
Pitavastatin 2 mg	779,926	24,484	6004	4.0	2.0	2.2	33.7	12.291	12.241	24,850	0.025	0.029	977,000	844,000
Scenario 3) Using Rama‐EGAT equation														
No statin	757,759	0	0	6.0	2.9	2.9	34.3	12.251	12.192	–	–	–	–	–
Pravastatin 20 mg	846,384	89,993	0	5.4	2.5	2.6	34.0	12.272	12.218	Dominated (extended)				
Pitavastatin 2 mg	853,007	97,396	0	5.3	2.4	2.5	33.9	12.276	12.223	95,248	0.024	0.031	3,902,000	3,090,000

Incremental cost‐effectiveness for each strategy was measured relative to the next best strategy in terms of QALYs gained. Pravastatin is dominated (extended) by pitavastatin as pravastatin has a higher ICER compared with no statin and is less effective than pitavastatin. Costs, QALYs, and life‐years were discounted at 3%/year. Costs can be converted to $US by dividing by 31.16. CVD, cardiovascular disease; ICER, incremental cost‐effectiveness ratio; MI, myocardial infarction; QALY, quality‐adjusted life‐year; Rama‐EGAT, Ramathibodi‐Electricity Generating Authority of Thailand.

a Per 1000 person‐years

b rounded to nearest thousand.

Pravastatin was estimated to be less effective and less cost‐effective than pitavastatin and was therefore dominated by extension (extended dominance) by pitavastatin (Figure S7). Compared with patients in the no statin group, patients receiving pitavastatin had 21.3%, 22.7% and 16.0% reductions in the incidence of MI, ischaemic stroke and fatal CVD, respectively, and accumulated 0.042 additional QALYs at an incremental cost of 96,442 Baht ($US3095), giving an ICER of 2,300,000 Baht ($US73,812)/QALY gained (Table 3).

### 3.2 Sensitivity analyses

**Figure 1:**
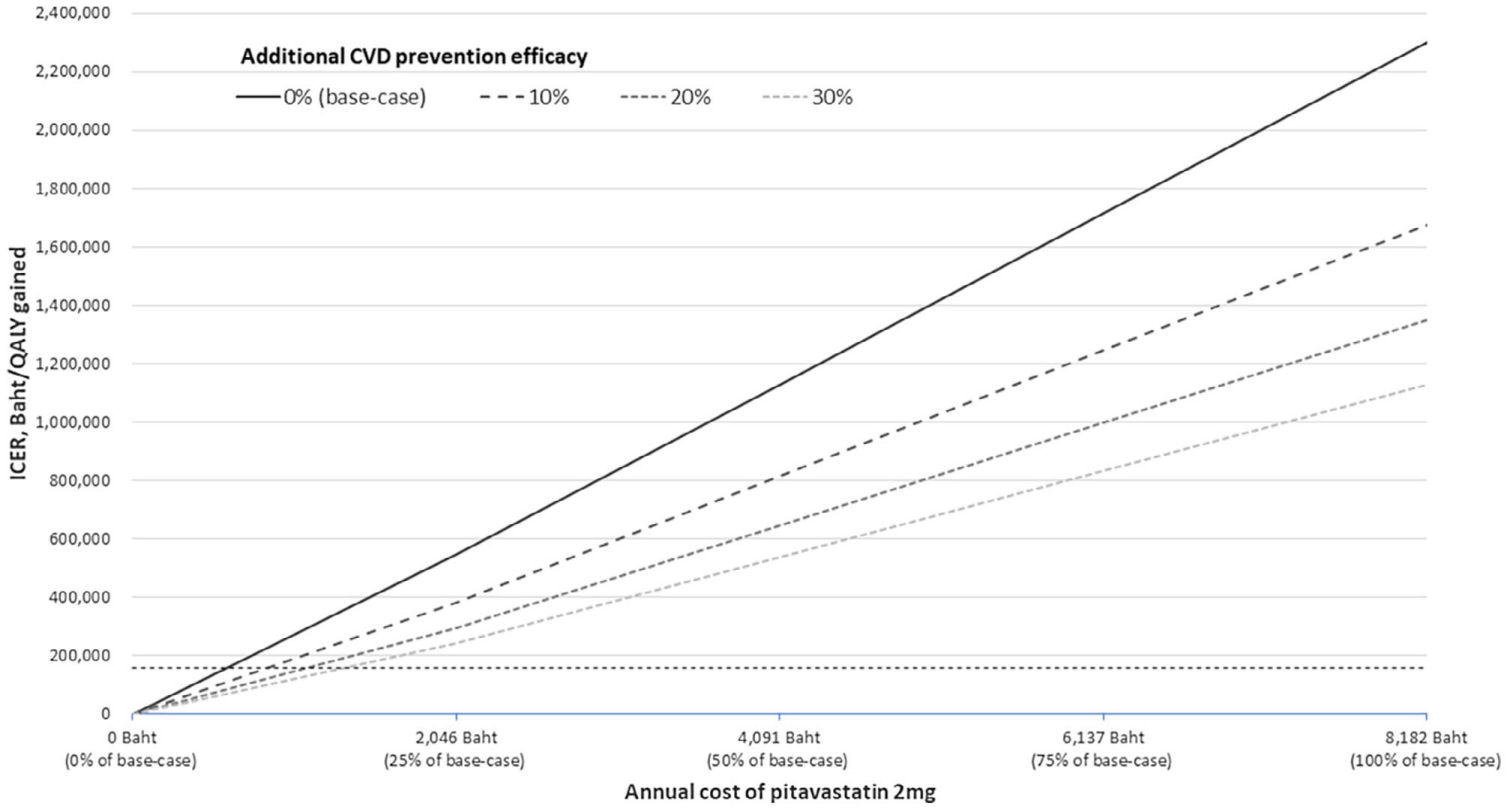
ICER for pitavastatin vs. no statin under various assumptions for pitavastatin cost and additional CVD prevention efficacy. ^†^The probability of CVD while using pitavastatin was reduced by various percentages to account for the possibility of preventative efficacy beyond cholesterol improvement in PLHIV; Horizontal dashed line represents a willingness‐to‐pay threshold of 160,000 Baht/QALY gained; Costs can be converted to $US by dividing by 31.16; ICER, incremental cost‐effectiveness ratio; CVD, cardiovascular disease; PLHIV, people living with HIV; QALY, quality‐adjusted life‐year.

Our one‐way sensitivity analysis results for pravastatin versus no statin and pitavastatin versus no statin are presented in full in Figures S8,S9 respectively. Base‐case findings were sensitive to changes in annual drug cost and drug efficacy. However, even at the lower end of our price ranges for pravastatin (3784 Baht ($US121.40)) and pitavastatin (4091 Baht ($US131.30)) neither was cost‐effective compared to no statin. At a willingness‐to‐pay threshold of 160,000 Baht ($US5,315)/QALY gained, the annual cost of pravastatin needed to drop to 415 Baht ($US13.30; 5.5% of base‐case price) to become cost‐effective compared to no statin, and the annual cost of pitavastatin needed to drop to 600 Baht ($US19.30; 7.3% of base‐case price) to become cost‐effective compared to no statin. When the probability of CVD while using a statin was reduced by 30% to account for the possibility of statins exhibiting CVD preventative efficacy in PLHIV beyond that associated with cholesterol improvement, the ICER for pitavastatin versus no statin improved to 1,130,000 Baht ($US36,264)/QALY gained. In a two‐way sensitivity analysis, where the probability of CVD while using pitavastatin was reduced by 30%, the annual cost of pitavastatin needed to drop to 1350 Baht ($US43.30; 16.5% of the base‐case price) to become cost‐effective compared with no statin (Figure 1).

In our base‐case analysis, we assumed that the pill burden associated with daily statin use did not cause any quality‐of‐life decrement. When a decrement was assumed, the average number of QALYs accumulated in the active treatment arms was reduced substantially. At the upper bound of our sensitivity range (0.00384 QALYs lost per year, the equivalent of losing four weeks of perfect health over 20 years [53]), both pravastatin and pitavastatin resulted in a net QALY loss compared to no statin use.

A time horizon longer than that used for the base‐case analysis resulted in more favourable ICERs for both the pravastatin versus no statin and pitavastatin versus no statin comparisons. For example when the time horizon was extended to 30 years, the pitavastatin versus no statin ICER improved to 1,530,000 Baht ($US49,101)/QALY gained.

**Figure 2:**
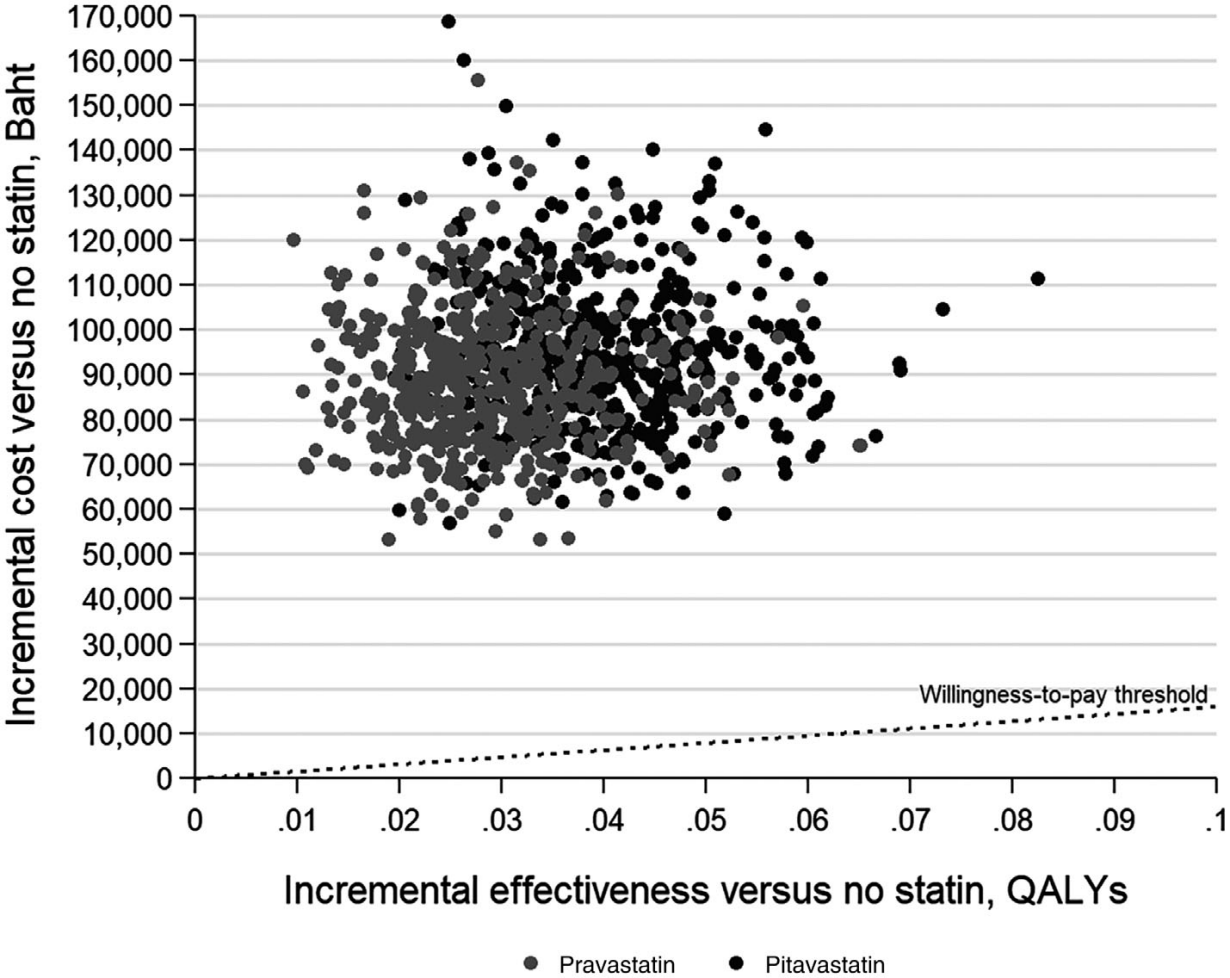
Probability sensitivity analysis scatter plot of incremental cost and incremental effectiveness for pravastatin and pitavastatin versus no statin. Willingness‐to‐pay threshold defined as 160,000 Baht/QALY gained; Costs can be converted to $US by dividing by 31.16; QALY, Quality‐adjusted life‐year.

In our probabilistic sensitivity analysis, pravastatin and pitavastatin were not cost‐effective in any simulation at a willingness‐to‐pay threshold of 160,000 Baht ($US5315)/QALY gained (Figure 2).

### 3.3 Scenario analyses

The results of our scenario analyses are displayed in Table 3. In all scenarios, pitavastatin remained dominant (extended) over pravastatin. Treating only patients at> 1% risk of CVD in the next five years (Scenario 1) slightly improved the ICER for pitavastatin versus no statin to 2,270,000 Baht ($US72,812)/QALY gained. Restricting statin therapy to only those at> 5% risk of CVD in the next five years (Scenario 2) further improved the ICER for pitavastatin versus no statin to 844,000 Baht ($US27,086)/QALY gained. The ICERs comparing pitavastatin versus no statin in Scenarios 1 and 2 improved marginally when we removed the costs associated with blood lipid testing (2,241,000 and 640,000 Baht ($US71,919 and $US20,539)/QALY gained respectively). When using the Rama‐EGAT equation in place of the D:A:D equation for estimating CVD risk (Scenario 3), our model predicted higher rates of MI, ischaemic stroke and fatal CVD compared with the base‐case. However, costs increased and the number of QALYs gained decreased compared with the base‐case (ICER for pitavastatin versus no statin, 3,090,000 Baht ($US99,166)/QALY gained).

## 4 Discussion

While expanding pravastatin or pitavastatin use to PLHIV not currently using lipid‐lowering therapy would help reduce the excess risk of CVD among PLHIV in Thailand, we estimated that neither option would be cost‐effective at current drug prices. Our results were sensitive to statin costs and statin efficacy, the burden associated with taking an additional daily pill, and the targeting of PLHIV based on CVD risk. However, our primary conclusions were robust across a wide range of sensitivity and scenario analyses.

Tamteerano *et al* estimated that generic simvastatin use for the primary prevention of CVD among all Thai adults with a 10‐year CVD risk> 2.5% would be cost‐effective at a willingness‐to‐pay threshold of 300,000 Baht ($US9,628)/QALY gained [17]. Similarly, Ribeiro *et al* found that intermediate potency statins (defined as those expected to produce a 30% to 40% reduction in LDL levels) would be cost‐effective for the primary prevention of CVD among those in the general population of Brazil with a 10‐year CVD risk greater than 5% [16]. There are several reasons our results differ for the HIV population in Thailand. There is a higher frequency of events competing with CVD in PLHIV compared with the general population. While HIV is an independent risk factor for CVD, the absolute burden of CVD death among PLHIV is lower than in the general population because PLHIV more frequently die from other causes [72, 73]. Therefore, preventing CVD among PLHIV results in fewer QALYs gained compared with preventing CVD in the general population. PLHIV also have higher background healthcare costs than the general population, and the abovementioned general population studies were able to assume a lower cost of statin use (for example, 296 Baht ($US9.50)/year for generic simvastatin in Tamteerano *et al*. [17]) than we did because of the low potential for drug interactions among the general population when using cheaper statins. Whether cheaper, non‐preferred statins could be a clinically acceptable alternative to pravastatin and pitavastatin in PLHIV at relatively low risk of CVD, or whether the current costs of pravastatin and pitavastatin could be reduced, are issues we are currently investigating (Box 1).

Box 1 **Further research questions**

**Table:** 

Do the anti‐inflammatory properties of statins reduce the probability of CVD in PLHIV beyond what is achievable with cholesterol improvement? Is there a quality‐of‐life decrement associated with taking an additional daily pill among PLHIV? To what extent can the current cost of pravastatin and pitavastatin in Thailand be reduced? Could cheaper, non‐preferred statins be a clinically acceptable alternative to pravastatin and pitavastatin in PLHIV at relatively low risk of CVD?

An important consistency between our study and that of earlier statin studies was the impact of including a quality‐of‐life decrement associated with pill burden [53, 74]. In our analysis, QALYs gained for pravastatin and pitavastatin quickly became negative compared with the no statin group when we included a small decrement in quality‐of‐life associated with remembering to take a daily statin and the inconvenience of doing so. Similarly, when a small pill burden was included in their general population model, Pandya *et al*. [74] found that the optimal CVD risk score threshold for statin indication increased three‐fold (from 5% to 15% 10‐year risk) at a willingness‐to‐pay of $US150,000/QALY gained. Current estimates of quality‐of‐life decrement associated with pill use vary widely [75, 76]. However, for PLHIV, who are well versed in the importance of good ART adherence, the burden of taking an additional daily pill is likely to be negligible (Box 1).

The average improvement in cholesterol associated with statin therapy leads to a 15% to 20% reduction in major CVD events [11]. It remains unknown whether the anti‐inflammatory properties of statins further reduce the probability of CVD in PLHIV. The Randomized Trial to Prevent Vascular Events in HIV (REPRIEVE) study is currently investigating pitavastatin for the primary prevention of CVD in PLHIV at low‐ to moderate‐risk of CVD [77]. This trial is expected to conclude in 2022 and will shed light on the overall CVD preventative efficacy of statins in PLHIV (Box 1). However, we have shown that even if there was an additional 30% decrease in the probability of CVD with statin use in PLHIV (on top of the reduced probability associated with cholesterol improvement) the cost of pitavastatin would still need to drop substantially before it became cost‐effective compared with no statin for the primary prevention of CVD in those not already using lipid‐lowering therapy. Importantly, REPRIEVE is also investigating the impact of statin use on various non‐CVD outcomes, including AIDS‐defining illness, non‐AIDS‐defining cancer, renal disease, and cirrhosis [77]. Although there is a paucity of literature supporting the benefit of statins in preventing non‐CVD events [63], such evidence could alter our main findings. It will also be useful to repeat our analysis in different settings as the protocols and costs associated with HIV and CVD in countries outside of Thailand are likely to differ substantially.

There are several limitations to this study. First, there is evidence suggesting that the D:A:D equation underestimates CVD risk among PLHIV. However, this is based on an analysis of the HIV Outpatient Study [78] which underestimates the prevalence of CVD family history – a key variable in the D:A:D equation. Furthermore, we found that our main findings were unchanged when we used the Rama‐EGAT equation to calculate MI and stroke risk. Second, although our model includes coronary intervention, MI, stroke and cardiovascular death as elements of CVD, we did not incorporate peripheral artery disease as it is not an outcome included in the D:A:D equation. Recent evidence suggests HIV infection is associated with a 19% increased risk of peripheral artery disease beyond that explained by traditional atherosclerotic risk factors [79]. Third, we had to estimate various model parameters using data from the general population or from high‐income settings due to a lack of HIV‐specific or resource‐limited setting data. While it is plausible that these parameters differ substantially between the general population and PLHIV, or between high‐income and resource‐limited settings, our sensitivity analyses suggested that this would have minimal impact on our main findings. Finally, we assumed that half doses of pravastatin and pitavastatin in Thai PLHIV would exhibit the same efficacy as typical doses used for non‐Asian PLHIV based on prior studies of other statins [62]. If, in fact, pravastatin 40 mg and pitavastatin 4 mg doses are more appropriate for Thai PLHIV, this would increase our cost estimates for both drugs making them less cost‐effective compared with no statin.

## 5 Conclusions

At a willingness‐to‐pay threshold of 160,000 Baht ($US5,315)/QALY gained, neither pravastatin nor pitavastatin were projected to be cost‐effective for the primary prevention of CVD among PLHIV in Thailand not currently using lipid‐lowering therapy (Box 2). These findings were sensitive to the targeting of PLHIV based on CVD risk, the burden associated with taking an additional daily pill, statin costs and statin efficacy. However, our primary conclusions were robust across a wide range of sensitivity and scenario analyses.

Box 2 **Policy implications of findings**

**Table:** 

At current drug prices, neither pravastatin nor pitavastatin are likely to be cost‐effective for the primary prevention of CVD among PLHIV in Thailand not currently using lipid‐lowering therapy.

## Competing interests

DCB has received research funding from Gilead Sciences and is supported by a National Health and Medical Research Council Early Career Fellowship (APP1140503); MGL has received unrestricted grants from Boehringer Ingelhiem, Gilead Sciences, Merck Sharp & Dohme, Bristol‐Myers Squibb, Janssen‐Cilag, and ViiV HealthCare and consultancy fees from Gilead Sciences and data and safety monitoring board sitting fees from Sirtex Pty Ltd; All other authors report no potential competing interests.

## Authors’ Contributions

DCB conceived of and carried out the analysis and drafted the manuscript; ATN, PC, AP, EB, MGL, JGK and SB provided critical input to the design of the analysis; RC, SKh, AA, SKi and JR were essential in the collection of patient data; PC and SKi made significant contributions to the parameterization of the model; SKi oversaw the project from start to finish; All authors helped draft the manuscript and have read and approved the final submission.

## Abbreviations

ART, Antiretroviral therapy; CABG, Coronary artery bypass graft; CVD, Cardiovascular disease; D:A:D, Data collection on Adverse Effects of Anti‐HIV Drugs; ICER, Incremental cost‐effectiveness ratio; MI, Myocardial infarction; PCI, Percutaneous coronary intervention; PLHIV, People living with HIV; QALY, Quality‐adjusted life‐year; Rama‐EGAT, Ramathibodi‐Electricity Generating Authority of Thailand; TAHOD, TREAT Asia HIV Observational Database.

## Supporting information


**Table S1.** Annual probability of developing diabetes by age and sex
**Table S2.** Annual probability of smoking cessation by age
**Table S3.** Annual increase in systolic blood pressure (mmHg) by age and sex^†^
**Figure S1.** Core model structure.
**Figure S2.** Probability of all‐cause death.
**Figure S3.** Probability of recurrent MI.
**Figure S4.** Probability of recurrent ischaemic stroke.
**Figure S5.** Probability of ischaemic stroke after MI.
**Figure S6.** Probability of MI after ischaemic stroke.
**Figure S7.** Cost‐effectiveness plane.
**Figure S8**. Tornado plot showing the impact of changes in model parameters on the incremental cost‐effectiveness ratio for pravastatin versus no statin.
**Figure S9.** Tornado plot showing the impact of changes in model parameters on the incremental cost‐effectiveness ratio for pitavastatin versus no statin.
