# Supplementary material for: Statins for atherosclerotic cardiovascular disease prevention in people living with HIV in Thailand: a cost‐effectiveness analysis
Source: J Int AIDS Soc. 2020 Jun 19;23(Suppl 1):e25494. doi: 10.1002/jia2.25494 (PMC7305414; doi:10.1002/jia2.25494)
Supplement: Supplementary file 1 — Table S1. Annual probability of developing diabetes by age and sex Table S2. Annual probability of smoking cessation by age Table S3. Annual increase in systolic blood pressure (mmHg) by age and sex† Figure S1. Core model structure. Figure S2. Probability of all‐cause death. Figure S3. Probability of recurrent MI. Figure S4. Probability of recurrent ischaemic stroke. Figure S5. Probability of ischaemic stroke after MI. Figure S6. Probability of MI after ischaemic stroke. Figure S7. Cost‐effectiveness plane. Figure S8. Tornado plot showing the impact of changes in model parameters on the incremental cost‐effectiveness ratio for pravastatin versus no statin. Figure S9. Tornado plot showing the impact of changes in model parameters on the incremental cost‐effectiveness ratio for pitavastatin versus no statin. [file JIA2-23-e25494-s001.docx]

**Supplementary material**

**Supplementary Figure 1 – Core model structure**

**

**

Individuals moved from the healthy state to the states shown above in annual cycles. The circles represent chance nodes where each branch to the right of the node is assigned a pre-specified probability. The unfilled triangles represent terminal nodes and indicate where an individual ends up at the conclusion of an annual cycle and the state in which they begin the next annual cycle. Filled triangles are absorbing states. Once in a disease state (MI, ischemic stroke, or hemorrhagic stroke), coronary intervention was removed from the decision tree; all other branches were as detailed above. Individuals accumulated costs and benefits up until their death or the time horizon, whichever came first. MI, myocardial infarction; CV, cardiovascular; PCI, percutaneous coronary intervention; CABG, coronary artery bypass graft.

**Supplementary Table 1 – Annual probability of developing diabetes by age and sex**

| **Age, years** | **Female** | **Male** |
| --- | --- | --- |
| 41-50 | 0·0072 | 0·0172 |
| >50 | 0·0158 | 0·0180 |

**Supplementary Table 2 – Annual probability of smoking cessation by age**

| **Age, years** | **Probability** |
| --- | --- |
| 40-54 | 0·113 |
| >54 | 0·172 |

**Supplementary Table 3 – Annual increase in systolic blood pressure (mmHg) by age and sex**†

| **Age, years** | **Female** | **Male** |
| --- | --- | --- |
| 40-43 | 1·37 | 0·74 |
| ≥44 | 1·89 | 1·02 |

†Annual increases in systolic blood pressure were only applied to patients not using antihypertensive medication

**Supplementary Figure 2 – Probability of all-cause death**

**
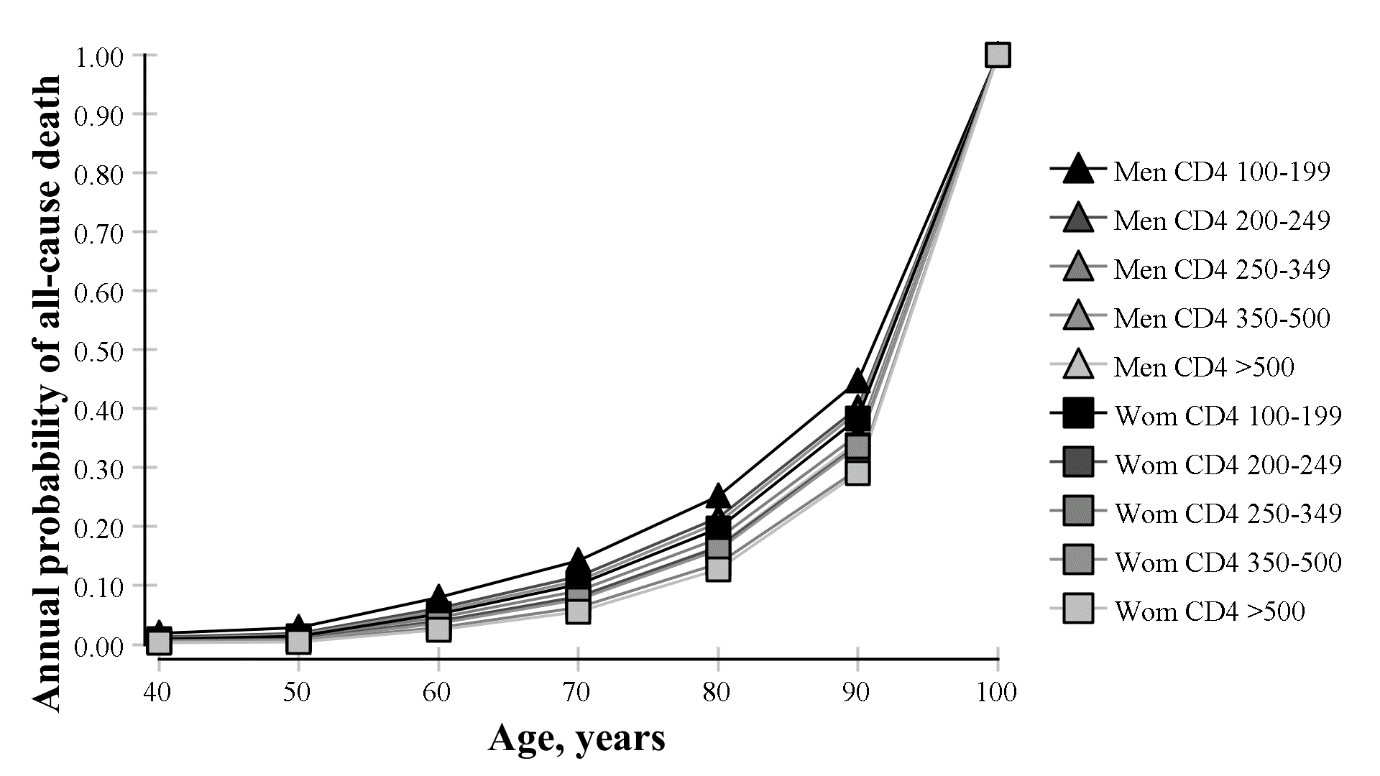
**

**Supplementary Figure 3 – Probability of recurrent MI**

**
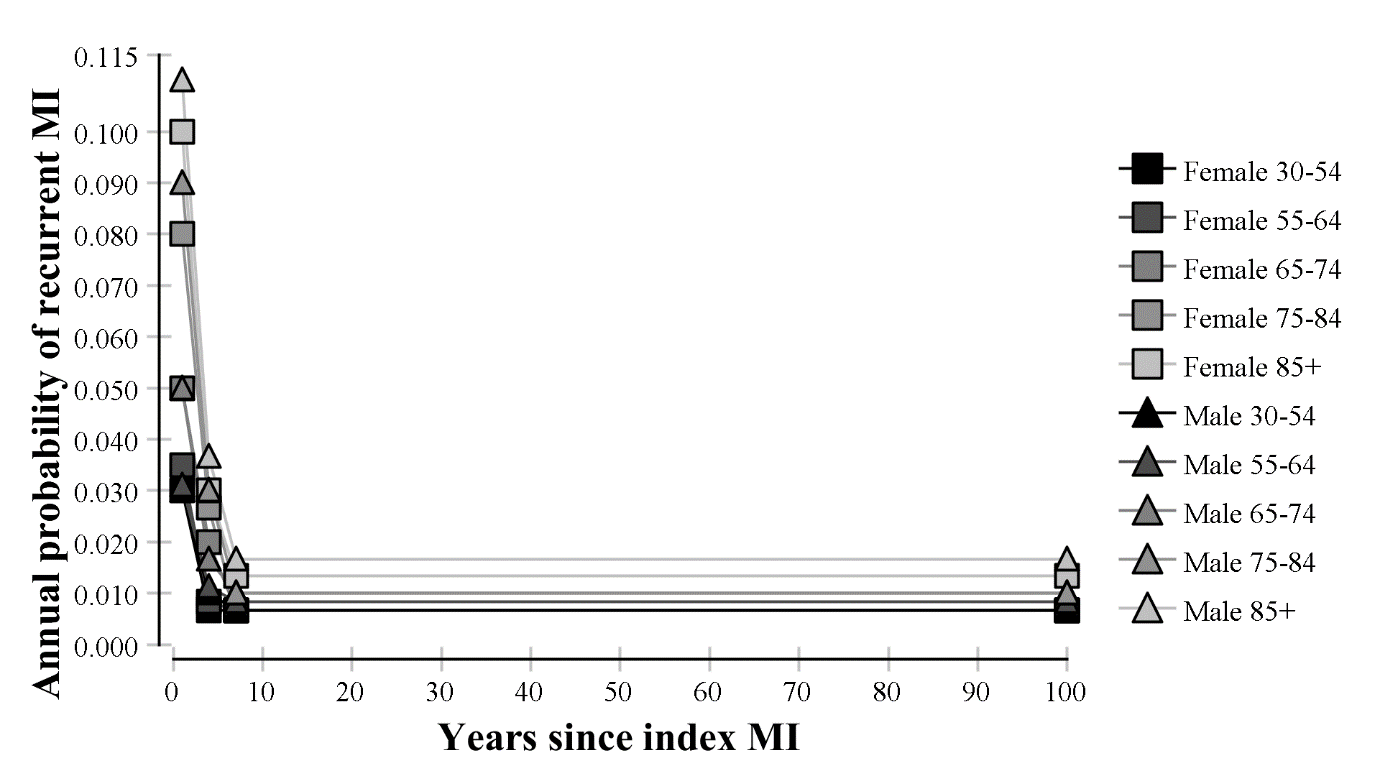
**

MI, myocardial infarction

**Supplementary Figure 4 – Probability of recurrent ischemic stroke**

**
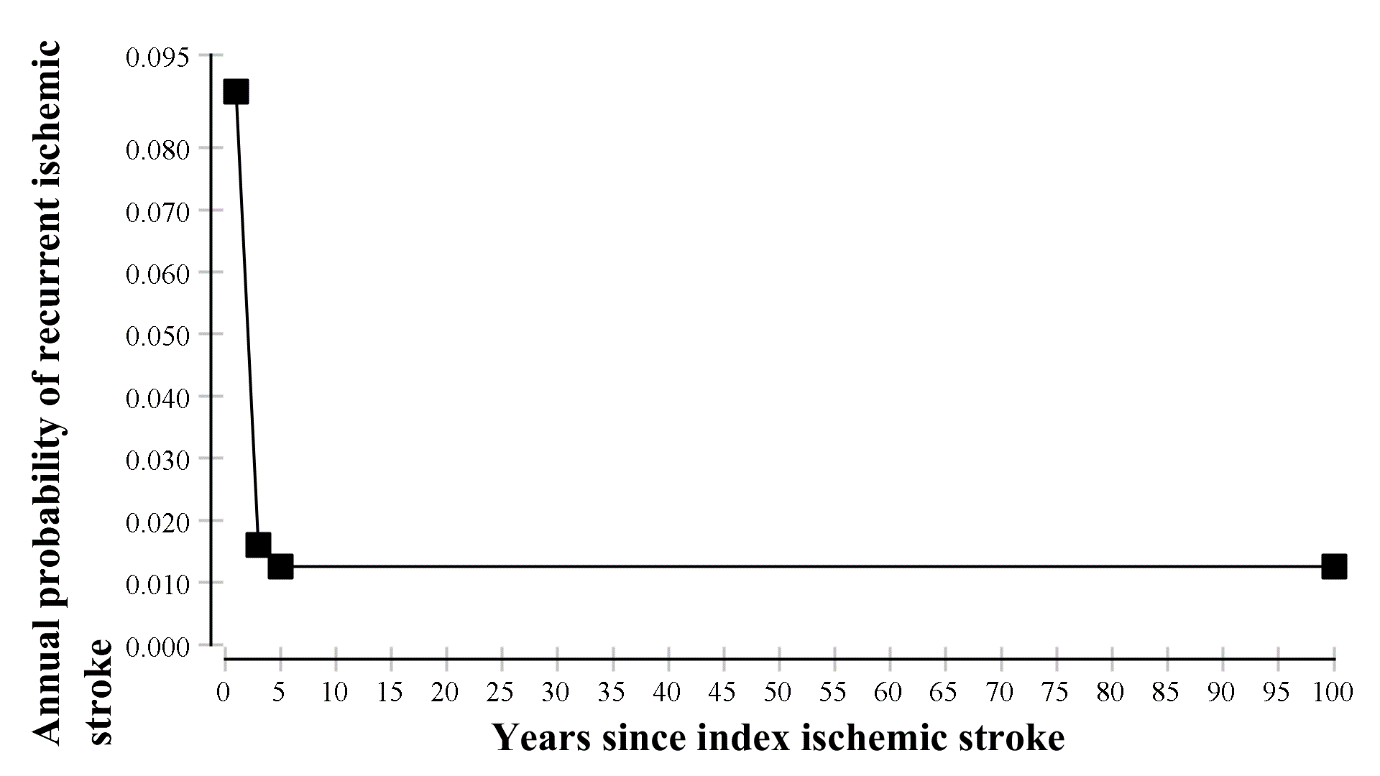
**

**Supplementary Figure 5 – Probability of ischemic stroke after MI**

**
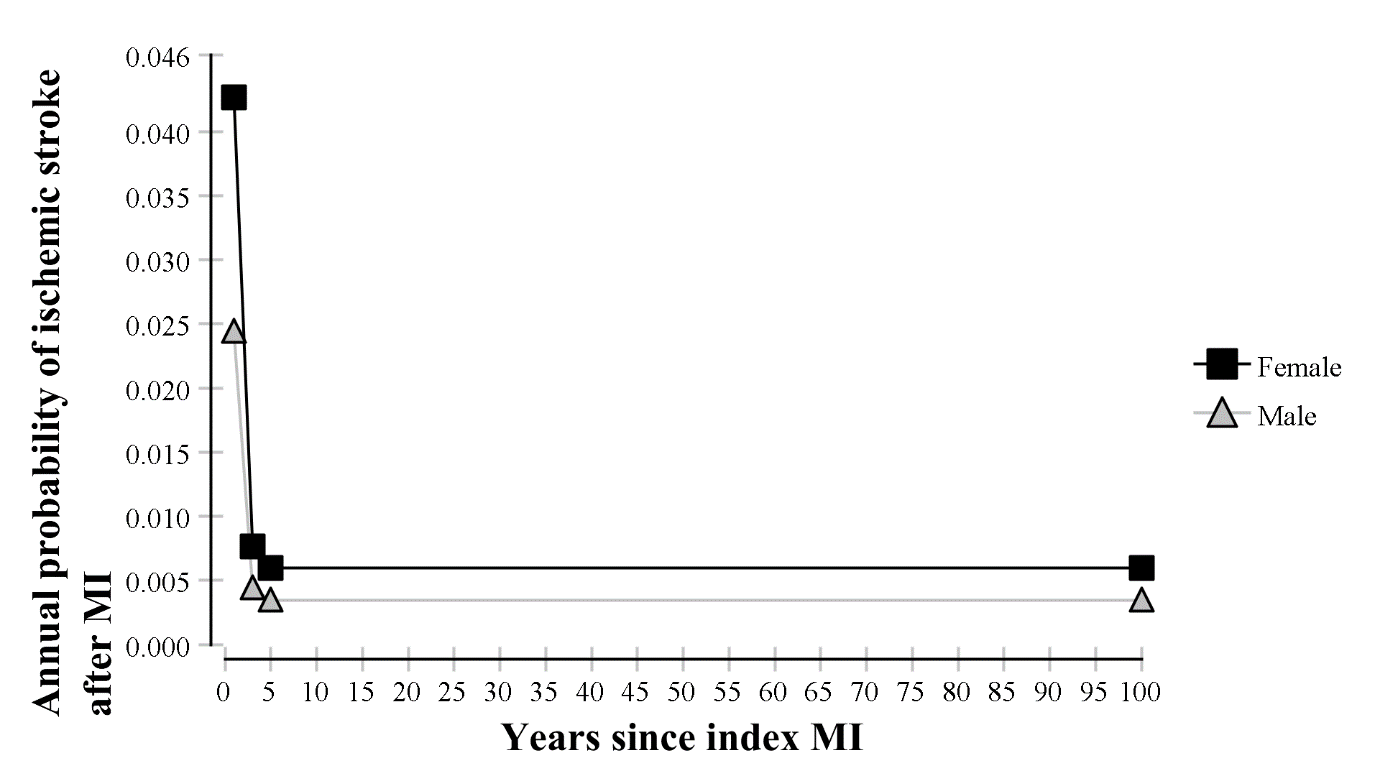
**

MI, myocardial infarction

**Supplementary Figure 6 – Probability of MI after ischemic stroke**

**
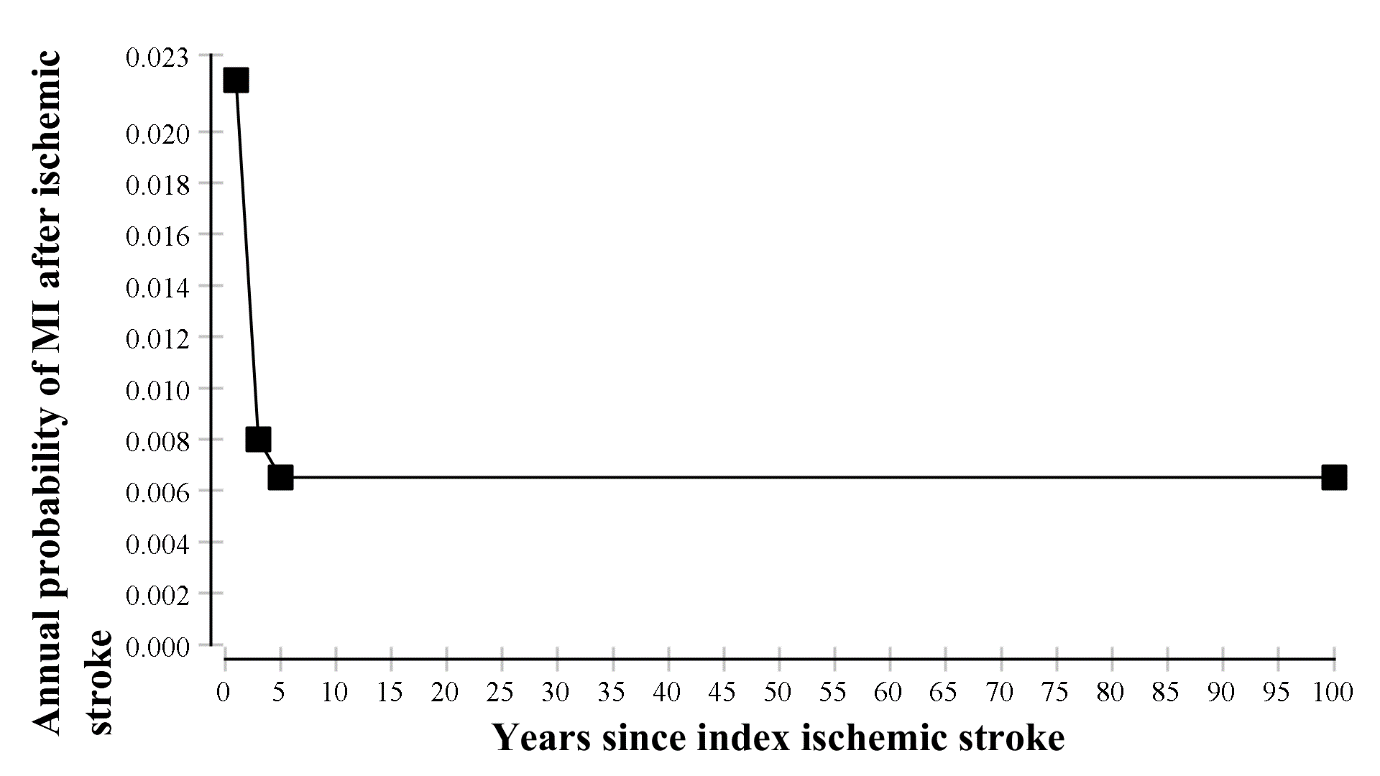
**

MI, myocardial infarction

**Supplementary Figure 7 – Cost-effectiveness plane**

**
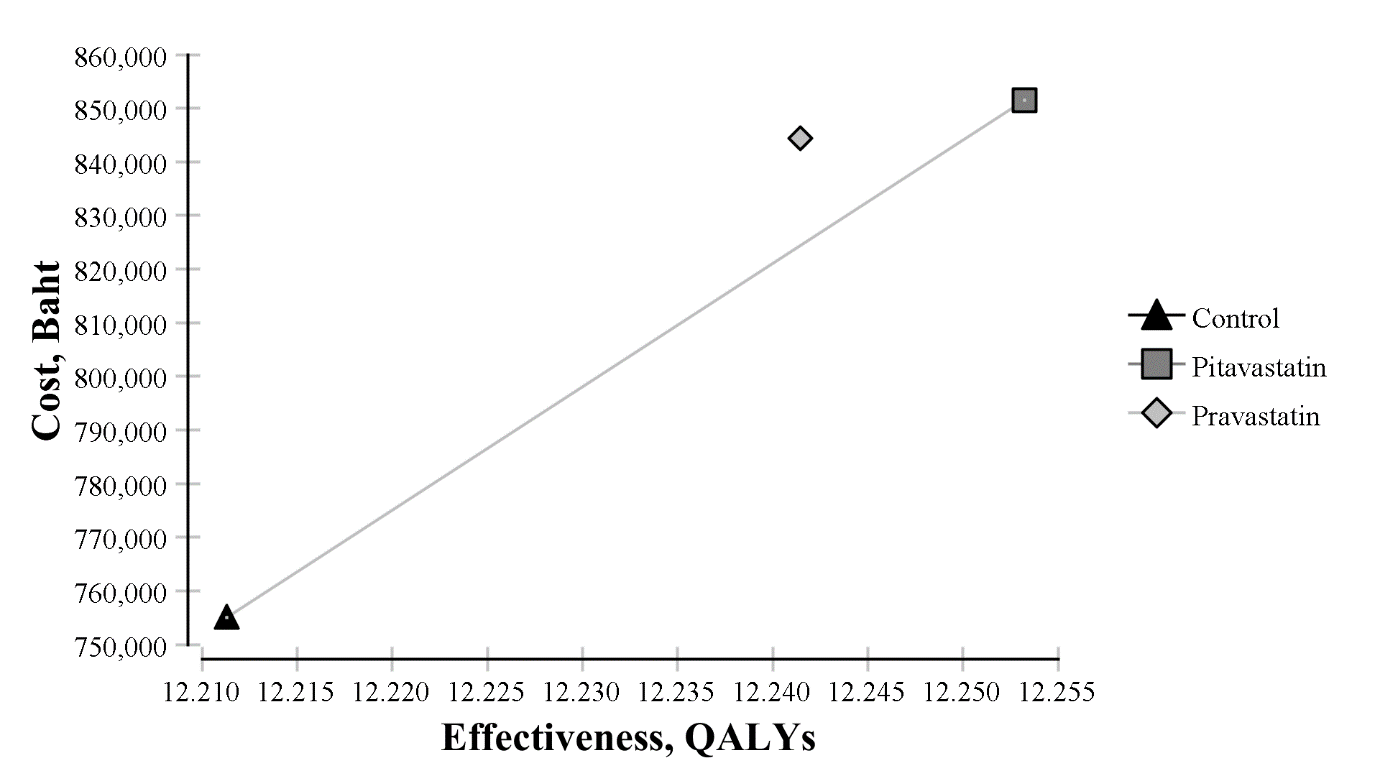
**

Pravastatin is dominated (extended) by pitavastatin as pravastatin has a higher ICER compared with no statin and is less effective than pitavastatin. Costs can be converted to $US by dividing by 31.16. QALY, quality-adjusted life-year; ICER, incremental cost-effectiveness ratio

**Supplementary Figure 8 – Tornado plot showing the impact of changes in model parameters on the incremental cost-effectiveness ratio for pravastatin versus no statin**

**
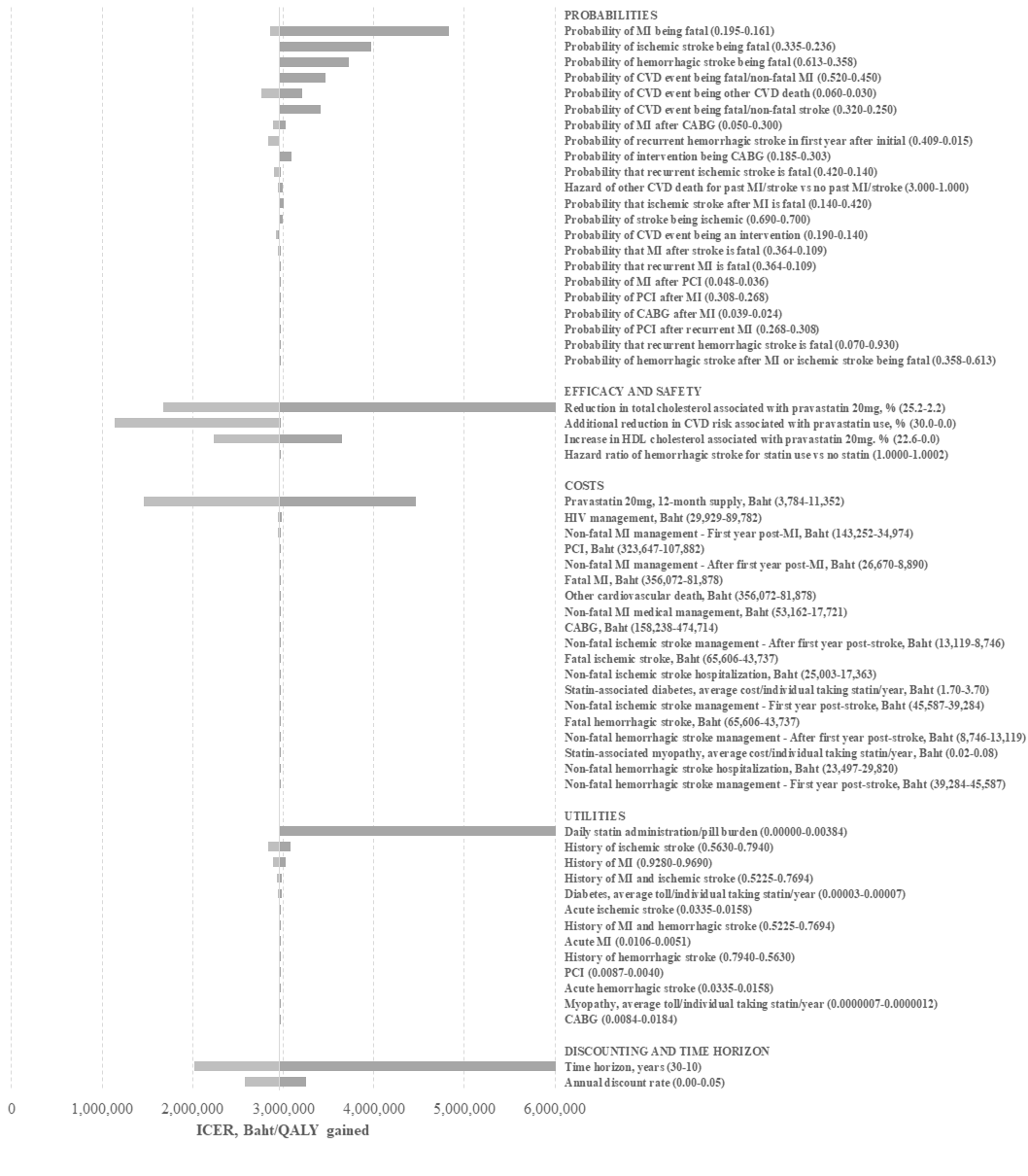
**

Costs can be converted to $US by dividing by 31.16. MI, myocardial infarction; CVD, Cardiovascular disease; CABG, coronary artery bypass graft; PCI, percutaneous coronary intervention; ICER, incremental cost-effectiveness ratio; QALY, quality-adjusted life-year

**Supplementary Figure 9 – Tornado plot showing the impact of changes in model parameters on the incremental cost-effectiveness ratio for pitavastatin versus no statin**

**
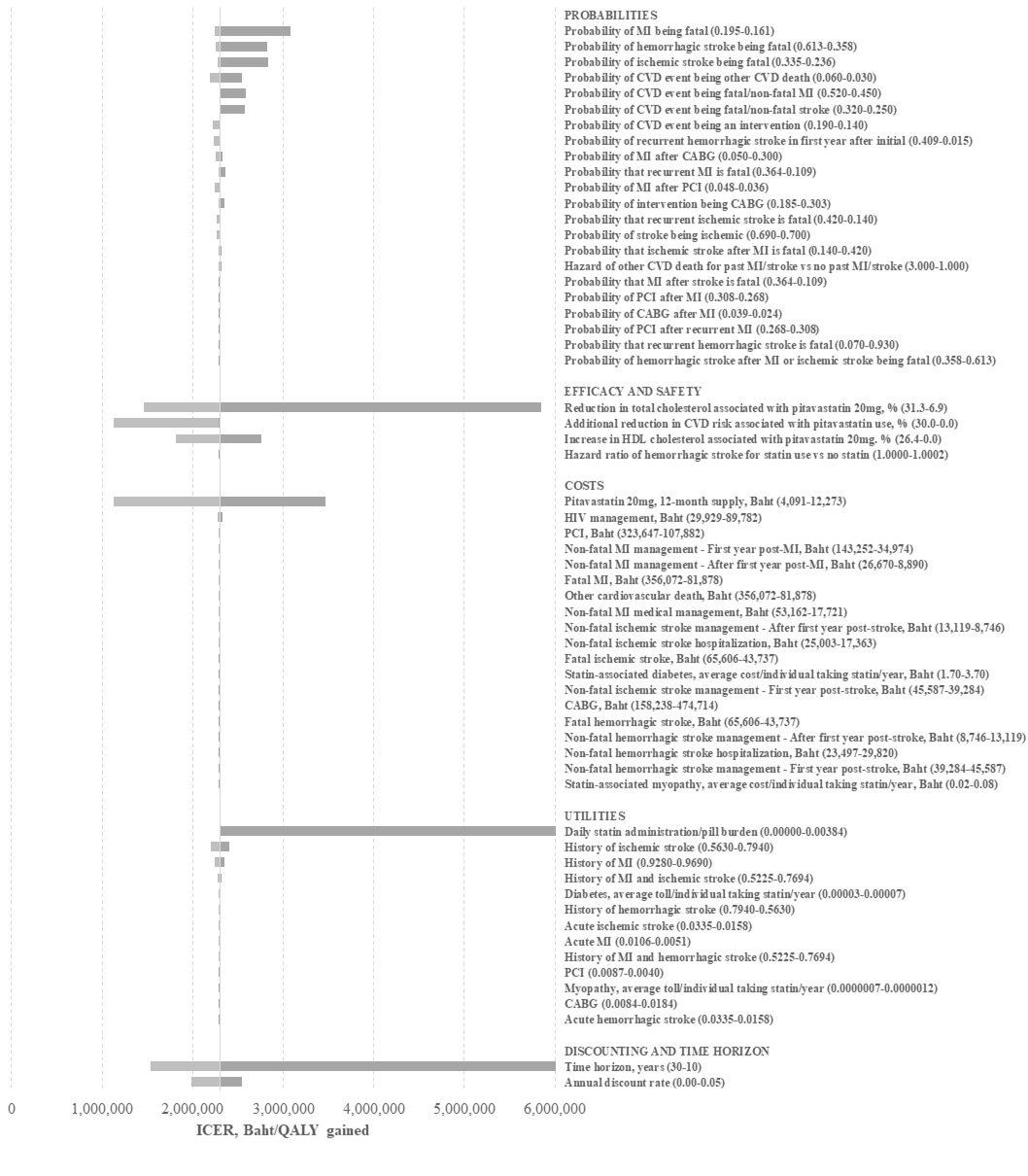
**

Costs can be converted to $US by dividing by 31.16. MI, myocardial infarction; CVD, Cardiovascular disease; CABG, coronary artery bypass graft; PCI, percutaneous coronary intervention; ICER, incremental cost-effectiveness ratio; QALY, quality-adjusted life-year
